# Impact of background parenchymal enhancement on the diagnosis of enhancing lesions in breast MRI: a systematic approach

**DOI:** 10.1007/s00330-026-12474-y

**Published:** 2026-04-04

**Authors:** Ambra Santonocito, Sonja Bechyna, Paola Clauser, Thomas H. Helbich, Pascal A. T. Baltzer

**Affiliations:** 1 High-field MR research center (HFMRC), Department of Biomedical Imaging and Image-guided Therapy, Medical University of Vienna, Vienna, Austria; 2 Department of Surgical Sciences, Paediatric Radiology Unit, University of Turin, Turin, Italy

**Keywords:** Breast cancer, Dynamic imaging, Magnetic resonance, Background parenchymal enhancement

## Abstract

**Objective:**

Dynamic contrast-enhanced MRI (DCE-MRI) enhances both suspicious lesions and normal tissue, known as background parenchymal enhancement (BPE). There is controversial evidence regarding whether BPE compromises the accuracy of breast MRI. Our aim was to investigate the influence of BPE in the detection of breast lesions by DCE-MRI using an ROI-based approach.

**Materials and methods:**

MRI Images of consecutive patients with indeterminate or suspicious findings on mammography or ultrasound (BI-RADS 0, 3, 4, 5). tested between May 2020 and September 2023 were retrospectively analyzed. Both breasts were divided into 10 regions of interest (ROIs), and BPE, BI-RADS and lesion type were assessed according to the MRI BI-RADS lexicon for each ROI by two blinded readers. BPE was dichotomized into minimum/mild (BPE1) and moderate/marked (BPE2). Patients without a standard reference were excluded. The standard reference was defined as histology or 1 year follow-up. The MRI performance was assessed using ROC analysis in an ROI-based analysis. Inter-reader agreement was assessed using k statistics.

**Results:**

A total of 316 patients (mean of 54 years, SD 10.6; range 31–84) were included. Among the 282 lesions histopathologically analyzed, 129 were malignant and 153 were benign. BPE1 was observed in 78% of patients and BPE2 in 22%. AUC did not differ between BPE1 (0.989) and BPE2 (0.988), *p* > 0.05. Sensitivity was 98.1% for BPE1 and 100% for BPE2, with comparable specificity: 95.5% (BPE1) vs. 92.6% (BPE2), *p* > 0.05.

**Conclusion:**

Our study demonstrated that BPE does not have a negative impact on the detection and identification of suspicious lesions on breast MRI.

**Key Points:**

***Question*** *What is the role of background parenchymal enhancement on DCE-MRI in terms of potential masking or mimicking remains a challenging issue?*

***Findings*** *The diagnostic accuracy of DCE-MRI for detecting breast lesions is not affected by elevated BPE levels.*

***Clinical relevance*** *Systematic evaluation of DCE-MRI using ROI-based analysis improves breast cancer detection and ensures diagnostic accuracy across different levels of background parenchymal enhancement.*

**Graphical Abstract:**

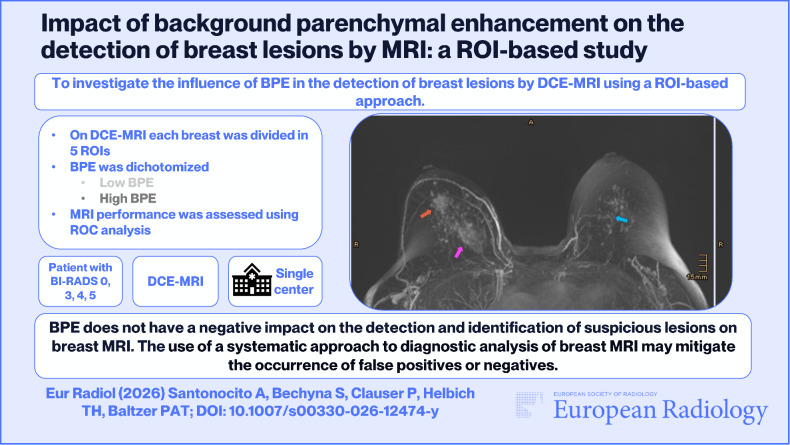

## Introduction

Dynamic contrast-enhanced magnetic resonance imaging (DCE-MRI) has the highest sensitivity for detecting breast cancer among current clinical imaging modalities [1]. However, after administration of intravenous contrast media, in addition to suspicious enhancing lesions on DCE-MRI, vessels, nipples, intramammary lymph nodes and normal fibroglandular parenchyma can also enhance. The enhancement of normal breast tissue is called background parenchymal enhancement (BPE) and has high variability over the course of the menstrual cycle and usually increases over the time course of dynamic scanning. Several physiological factors, like the expression of vascular endothelial growth factor and the density of blood vessels in the breast tissue, may impact BPE [2]. BPE is also influenced by steroid hormone levels [3] (i. e., estrogen and progesterone hormone levels). According to the MRI BI-RADS, BPE can be described as minimal (< 25% of glandular tissue showing enhancement), mild (25–50% enhancement), moderate (50–75% enhancement), or marked (> 75% enhancement) [4]. BPE typically presents as a peripheral pattern due to the blood inflow from the branches of the internal mammary and lateral thoracic arteries into the breast tissue. However, in some patients, it may manifest in other patterns, including central, nodular, regional, and diffuse foci of enhancement [5]. The ACR BI-RADS® MRI recommends assessing BPE for all patients on the first postcontrast subtraction image and to describe whether the pattern is asymmetric or symmetric [6].

While there seems to be only a weak positive correlation between the degree of background enhancement on DCE-MR images and radiographic breast density [7–9], a rapid and strong BPE could have a masking effect on MR images in analogy to a dense breast on mammography [10] and thereby hamper breast cancer detection.

There is controversial evidence regarding whether BPE compromises the accuracy of breast DCE-MRI by masking or mimicking suspicious enhancements. Several studies [11–14] demonstrated a higher abnormal interpretation rate for women with moderate or marked BPE, but with no significant impact on final diagnostic performance outcomes. Otherwise, some studies [15–17] demonstrated that higher BPE levels were directly related to diagnostic accuracy.

This study aimed to investigate the impact of BPE on the detection of breast lesions with DCE-MRI, applying an ROI-based approach. An ROI-based approach enables lesion-level assessment, accounting for the coexistence of benign and malignant findings within the same patient and providing a more practical measure of diagnostic performance than patient-based analyses.

## Materials and methods

This single-center, retrospective, observational study was approved by the Institutional Review Board (IRB), and the need for written informed consent was waived.

Images of consecutive patient that underwent MRI in line with ACR/EUSOBI recommendations between May 2020 and September 2023 were analyzed. The study included women with non-specific or suspicious findings identified through screening or diagnostic settings on mammography or ultrasound (BI-RADS 0, 3, 4, 5).

Patients without a standard reference, with renal insufficiency, or a history of allergy to gadolinium contrast media were not included. Moreover, patients who had undergone chemotherapy or antihormonal treatment, who were pregnant or breastfeeding, or who were not sent for MRI were excluded from the study. The standard reference was defined as histology obtained by image-guided biopsy or final surgical histology for suspicious lesions or a 12-month follow-up for non-suspicious ones.

### Breast magnetic resonance

Breast DCE-MRI was performed on either 1.5-T or 3-T scanners, with dedicated breast coils and patients in prone position. All protocols were multiparametric, and they included a T2-weighted sequence and a T1-weighted series acquired before and after injection of a gadolinium-based contrast agent, in accordance with international guidelines and recommendations. The temporal resolution was approximately 1 min (with minor variation of a few seconds), which is consistent with current international guidelines (e.g., EUSOBI, ACR, EUSOMA). A single-shot diffusion-weighted echo planar imaging (EPI) sequence (DWI) at b 0 and 800 s/mm^2^ was also included.

The ADC maps used for evaluation were automatically generated by the scanner software using a mono-exponential fit of the high and low b data.

### Image analysis

The images were assessed independently by two readers—one board-certified radiologist with three years of experience in breast imaging and an inexperienced reader (PhD candidate). The PhD student was a graduated medical doctor with previous training in radiology and particularly in breast radiology. Before starting the readings of this study, the PhD student underwent a 6-month intensive breast MRI fellowship training with three internationally renowned specialists in breast MRI, each with an experience of at least 16 years. Readers were blinded to the patient’s clinical data and histopathological results; moreover, they were blinded to whether other techniques were applied in the clinical workflow and to their findings. Evaluations were performed on dedicated workstations. Readers assessed the amount of fibroglandular tissue on T1-weighted native sequences and background parenchymal enhancement (BPE) on the first T1-weighted postcontrast fat-subtraction image. BPE was assessed on early enhanced series, strictly according to the definitions in the ACR BI-RADS lexicon. This time point was achieved when the inner parasternal vein was first contrasted. Readers assigned one of the four suggested levels on the original MRI images to ensure standardized interpretation. MIP reconstructions were available and reviewed together with the original images if needed.

**Fig. 1:**
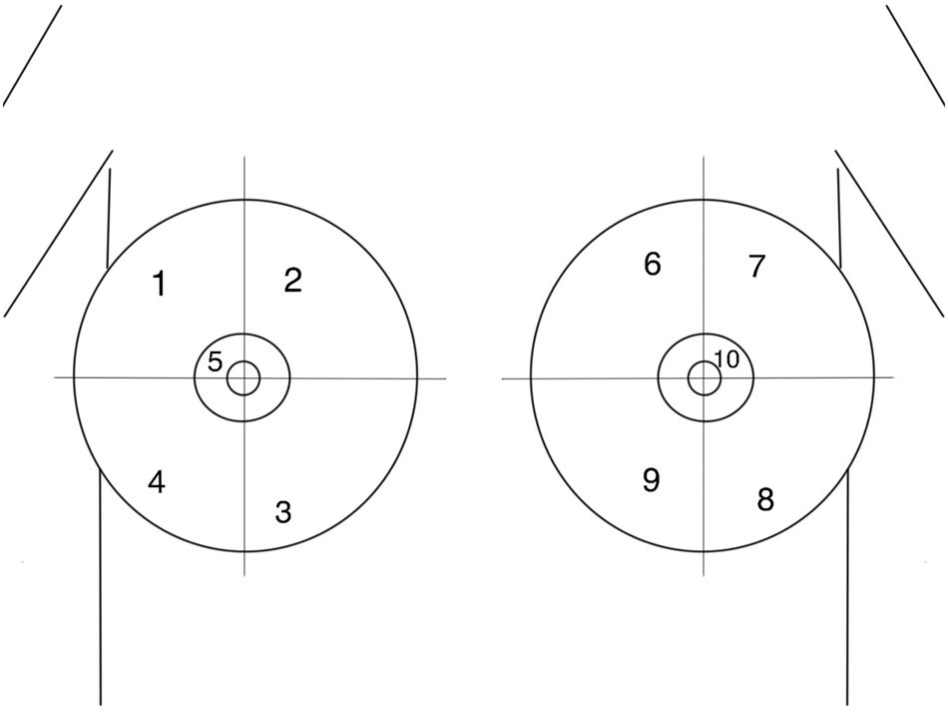
Graphical representation of the evaluation of the ROIs per patient. The ROIs are numbered as follows: craniolateral (1, 7); craniomedial (2, 6); caudolateral (4, 8); caudomedial (3, 9); and retro-areolar (5, 10)

Diagnostic accuracy was assessed using a region-based analysis framework as outlined by Obuchowski et al [18]. Each breast was divided into predefined volumetric regions that were assessed across the full dataset. Both breasts were divided into 5 volumetric regions of interest (ROI) defined as craniolateral, caudolateral, craniomedial, caudomedial, and retro-areolar, resulting in a total of 10 regions per patient (Fig. 1). Readers were required to both correctly localize and correctly classify lesions within these regions. Each ROI was therefore scored 1 (positive) if an enhancing lesion was present and 0 (negative) if no enhancing lesion was present, after which BI-RADS characterization was applied. For regions without enhancement, BI-RADS 1 or 2 was assigned depending on the presence of non-enhancing findings. For enhancing lesions, localization was performed within the respective volumetric region and categorized according to BI-RADS 2–5.

The age of the patient was recorded and categorized according to their menopausal status. Women younger than 44 years old were categorized as premenopausal, those with an age ranging between 45 and 55 years old were categorized as perimenopausal, and women older than 55 years old were categorized as postmenopausal.

In order to evaluate a lesion, readers were required to assess BI-RADS and enhancement type characteristics (mass, non-mass, mass/non-mass enhancement) in accordance with the BI-RADS lexicon for MRI. Readers assessed each region for enhancing lesions, which were then scored according to the BI-RADS system, using the Kaiser score decision rule if needed.

### Statistical analysis

Statistical analysis was conducted using Med-Calc and SPSS. The power analysis calculation was performed, assuming a two-sided α error of 5% and a β error of 10%, corresponding to a statistical power of 90%. The performance of MRI was assessed using the ordinal BI-RADS category assignments in an ROC analysis to determine the area under the ROC curve (AUC) as a general measure of diagnostic accuracy. The outcome was used as the classification variable. For the statistical analysis, dichotomized BPE groups were used, categorized as minimum/mild (BPE1) and moderate/marked (BPE2). This choice was based on prior research showing that the four categories are statistically less useful than a dichotomization between high and low BPEs [19]. Subgroup analyses utilized ordinal BPE ratings, and AUCs were recalculated and compared per subgroup. Sensitivity, specificity, and positive and negative likelihood ratios were calculated. The study was powered to detect at least AUC differences of 15% in a region-based analysis with a negative to positive case ratio of 20 and alpha and beta errors set to 5% and 20%, respectively. The correlation between BPE levels and menopausal status was made by using the Spearman correlation. The cancer prevalence was assessed for dichotomized BPE groups. *p*-values < 0.05 were considered statistically significant. The inter-reader agreement for BPE classification for ordinal and dichotomized variables (low = minimal/mild, high = moderate/marked) was evaluated using the weighted Kappa statistic.

## Results

**Fig. 2:**
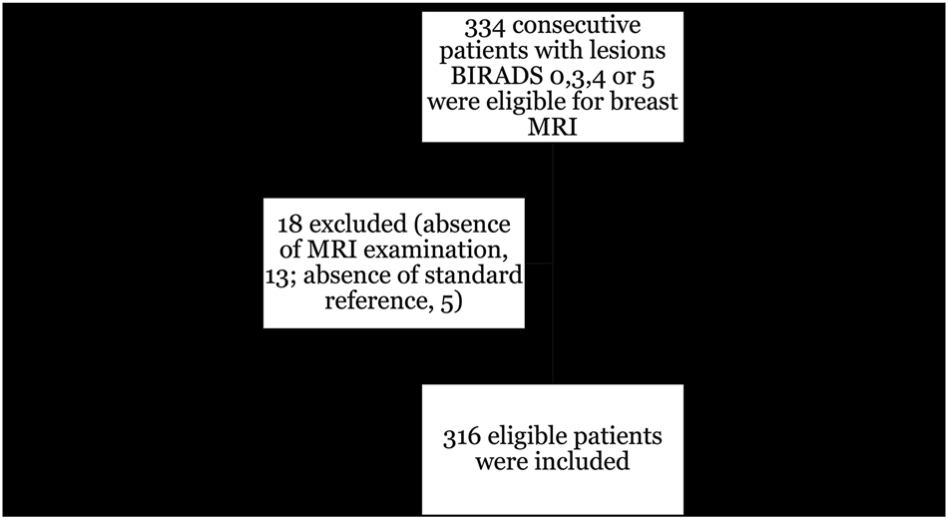
Study population flowchart

A total of 3160 regions of 316 patients were included in this ROI-based analysis. The mean age was 54 years (SD 10.6; range 31–84 years) (Fig. 2). Out of the 282 lesions histopathologically analyzed, 129 were malignant and 153 were benign. The classification of malignant and benign lesions is summarized in Table 1.

**Table 1:** Pathological analysis of the 282 breast lesions that underwent histological verification

	Histotype	*N*	%
Malignant lesions (129)	DCIS	32	24.8%
	ICD	84	65.1%
	ILC	7	5.4%
	Others	6	4.7%
Benign lesions (153)	Fibroadenoma	11	7.2%
	Adenosis	15	9.8%
	Adipose necrosis	7	4.6%
	Mastitis	16	10.5%
	B3	46	30.1%
	Hyperplasia/metaplasia	28	18.3%
	Granuloma	2	1.3%
	Radial scar	1	0.7%
	Breast parenchyma	14	9.2%
	Others	13	8.5%

**Fig. 3:**
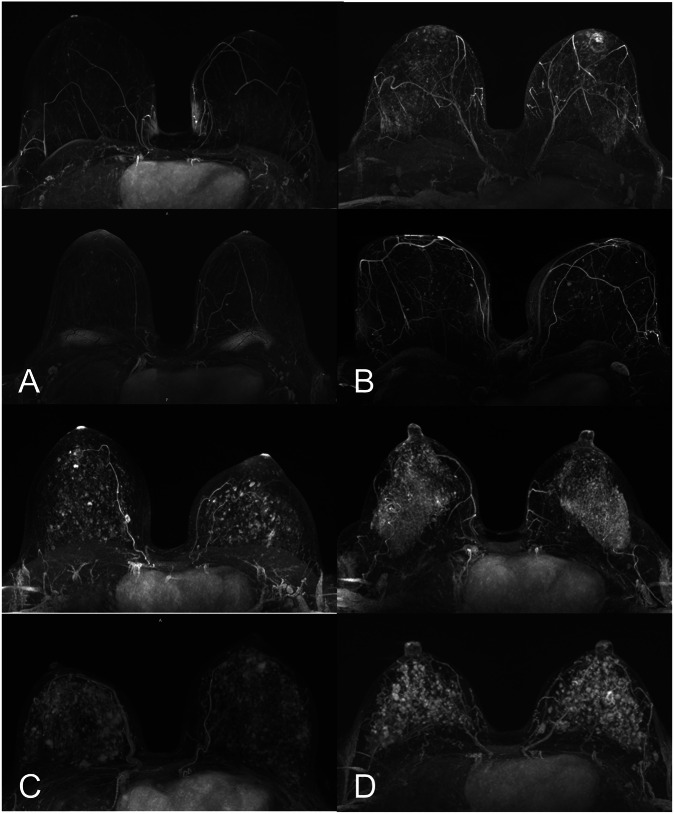
Two case examples for different levels of BPE according to the ACR: **A** (minimal), **B** (mild), **C** (moderate), **D** (marked). For simple representation, maximum intensity projections (MIPs) are displayed, though BPE should be assessed on early enhanced original images

Breast composition resulted as dense (ACR C and D) in 62% of patients. The agreement between the readers was substantial (k = 0.709). BPE was minimal in 50.9%, mild in 27.3%, moderate in 17.1%, and marked in 4.7% of patients (Fig. 3). BPE1 was observed in 249 patients and BPE2 in 68 patients. Inter-reader agreement was almost substantial for ordinal variables (k = 0.585) and moderate (k = 0.492) for dichotomized variables. Overall cancer prevalence was 45.7% (129/282). In the BPE1 group, the cancer prevalence was 46.7% (105/225), and in the BPE2 group was 42.1% (24/57) without a significant statistical difference (*p* > 0.05). Spearman correlation found a negative correlation between menopausal status and BPE levels (r = −0.191, *p* < 0.001).

To detect a clinically relevant difference of 10% in the AUC—for example, 95% versus 85%—a sample size of 64 cases per group was required. The area under the ROC curve (Supplementary Fig. 1) did not differ between BPE1 (0.989) and BPE2 (0.988), *p* > 0.05. Sensitivity of MRI was 98.1% in patients with BPE1 and 100% with BPE2, while specificity was nearly the same with BPE1 95.5% and BPE2 92.6%, *p* > 0.05. The demographics and radiological characteristics are summarized in Table 2.

**Table 2:** Demographics and radiological characteristics per regions of interest

	*N*	% or SD
Number of cases (region 1–10)	3160	
Number of patients	316	
Age	54	10.6
Menopausal status		
Premenopausal (< 44)	59	18.7%
Perimenopausal (45–55)	130	41.1%
Postmenopausal (> 56)	127	40.2%
Previous breast operations		
Yes	56	17.7%
No	260	82.3%
ACR categories		
A	230	7.3%
B	940	29.7%
C	1558	49.3%
D	432	13.7%
BPE level		
Minimal	1608	50.9%
Mild	863	27.3%
Moderate	539	17.1%
Marked	150	4.7%
BI-RADS categories		
1	2589	81.9%
2	288	9.1%
3	94	3.0%
4	128	4.1%
5	61	1.9%
Histology		
Benign	129	4.1
Malignant	3031	95.9

Mass enhancement was detected in 55.0% of malignant lesions 52.9% of benign lesions. Non-mass enhancement was detected in 34.9% of malignant lesions and in 26.8% of benign lesions. The average size was 23.2 mm (SD 20.6 mm) for malignant lesions, with the smallest lesion of 4 mm; and 16.7 mm (SD 14.9 mm) for benign ones, with the smallest lesion of 3 mm. The lesion characteristics stratified by the histology are summarized in Table 3.

**Table 3:** Lesion characteristics stratified by the histology

	Malignant lesions		Benign lesions	
	*N*	% or SD	*N*	% or SD
Number of lesions	129	45.7%	153	54.3%
Lesion size (mm)	23.2	20.6	16.7	14.9
Enhacement type				
Mass	71	55.0%	81	52.9%
Non-mass	45	34.9%	41	26.8%
Mass/non-mass	10	7.8%	1	0.7%
No enhancement	3	2.3%	30	19.6%

## Discussion

**Fig. 4:**
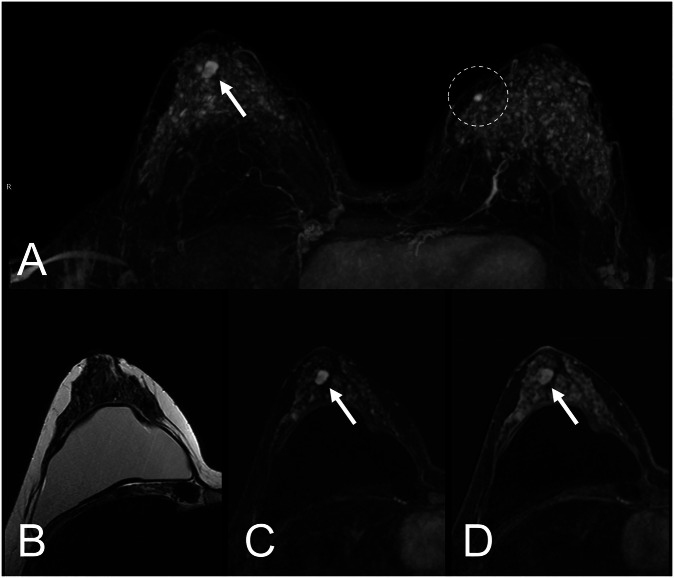
42-year old patient with dense breasts (ACR D) and marked bilateral BPE. The lesion (arrow) is visible on the MIP (**A**) and T2w (**B**) as well as early (**C**) and late (**D**) contrast-enhanced subtractions. The lesion is located in ROI 5, and it presents as an ill-defined mass with strong initial enhancement and heterogeneous wash-out, corresponding to a Kaiser score of 8 (highly suspicious). Targeted ultrasound-guided biopsy revealed invasive breast cancer NST G3. Note that a small benign lesion < 5 mm is visible also on the MIP (dashed circle in **A**)

Our study demonstrated that background parenchymal enhancement (BPE) did not affect the accuracy of DCE-MRI in detecting breast lesions, with high sensitivity and specificity (Fig. 4). Our results showed a negligible AUC difference of 0.1% between low and high BPE groups, suggesting a priori that there is little difference in diagnostic performance between the two groups.

**Fig. 5:**
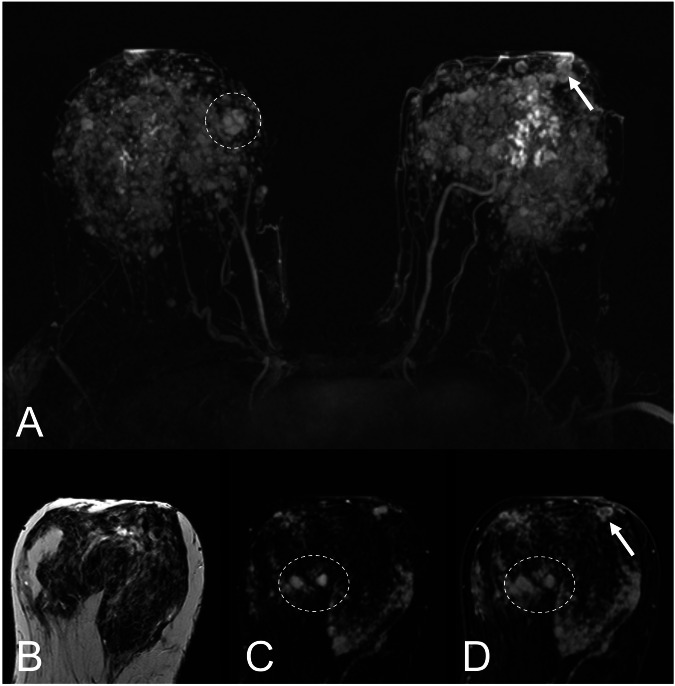
34-year-old patient with dense breasts (ACR D) and marked bilateral BPE. The lesion (arrow) is not well visible on the MIP (**A**) and T2w (**B**), while it can be seen well on early (**C**) and late (**D**) contrast-enhanced subtractions. The lesion presents as an oval rather circumscribed mass with central Wash-out, corresponding to a Kaiser score of 4 (low level of suspicion). Discharge could be provoked, and associated ducts were visible (not shown). Diagnosis was BI-RADS 4a (suspected papilloma) with targeted ultrasound-guided biopsy revealing papilloma without atypia. Note that several benign lesions are visible (dashed circle), partly also on the MIP (**A**)

It supports the high reliability and performance of DCE-MRI in clinical practice, indicating that it can be used with confidence to diagnose breast cancer in different patient populations (e.g., with moderate and marked BPE) without a significant impact on diagnostic accuracy. Indeed, in the present study, two benign lesions—one 5-mm intraductal papilloma (B3) and one 7-mm area of adenosis (B2)—occurring in patients with marked background parenchymal enhancement (BPE) were incorrectly classified as BI-RADS 4 (Fig. 5). Additionally, two other areas of marked BPE were overinterpreted as suspicious. It is important to note that no malignant lesions were misdiagnosed in patients with marked BPE, and among those with moderate BPE, only one case of ductal carcinoma in situ (DCIS) was underestimated as BI-RADS 3. While these findings highlight the potential for BPE to mimic or obscure malignant lesions, they also emphasize the possibility that a systematic approach to diagnostic analysis of breast MRI may mitigate the occurrence of false positives or negatives.

This finding is of clinical relevance, as there are divergent opinions regarding the impact of BPE on the MRI diagnostic performance. Previous studies have indicated that moderate or marked BPE may have an impact on specificity [11, 12, 20] and that BPE levels higher than minimal BPE may be associated with reduced accuracy [13, 17, 21, 22]. However, some other studies have shown no significant difference among different BPE levels in the biopsy rate and in the cancer detection rate [11, 23]. A recently published meta-analysis suggested BPE levels may have an impact on breast MRI diagnostic performance [19]. This meta-analysis comprised a limited number of studies that reported an apparent dependence of pooled diagnostic performance metrics on BPE. Its findings were limited by substantial between-study heterogeneity, which was not clarified by meta-regression—likely due to missing information regarding reading criteria, reader expertise, and study design across the included works. Furthermore, no study in that analysis employed a comprehensive region-based evaluation, leaving an important methodological gap.

Our study, employing both a structured approach with independent assessment of all regions and defined reading criteria (MRI lexicon and optionally the Kaiser score), was conceived in part to address these limitations and therefore contributes additional, much-needed evidence to this topic.

**Fig. 6:**
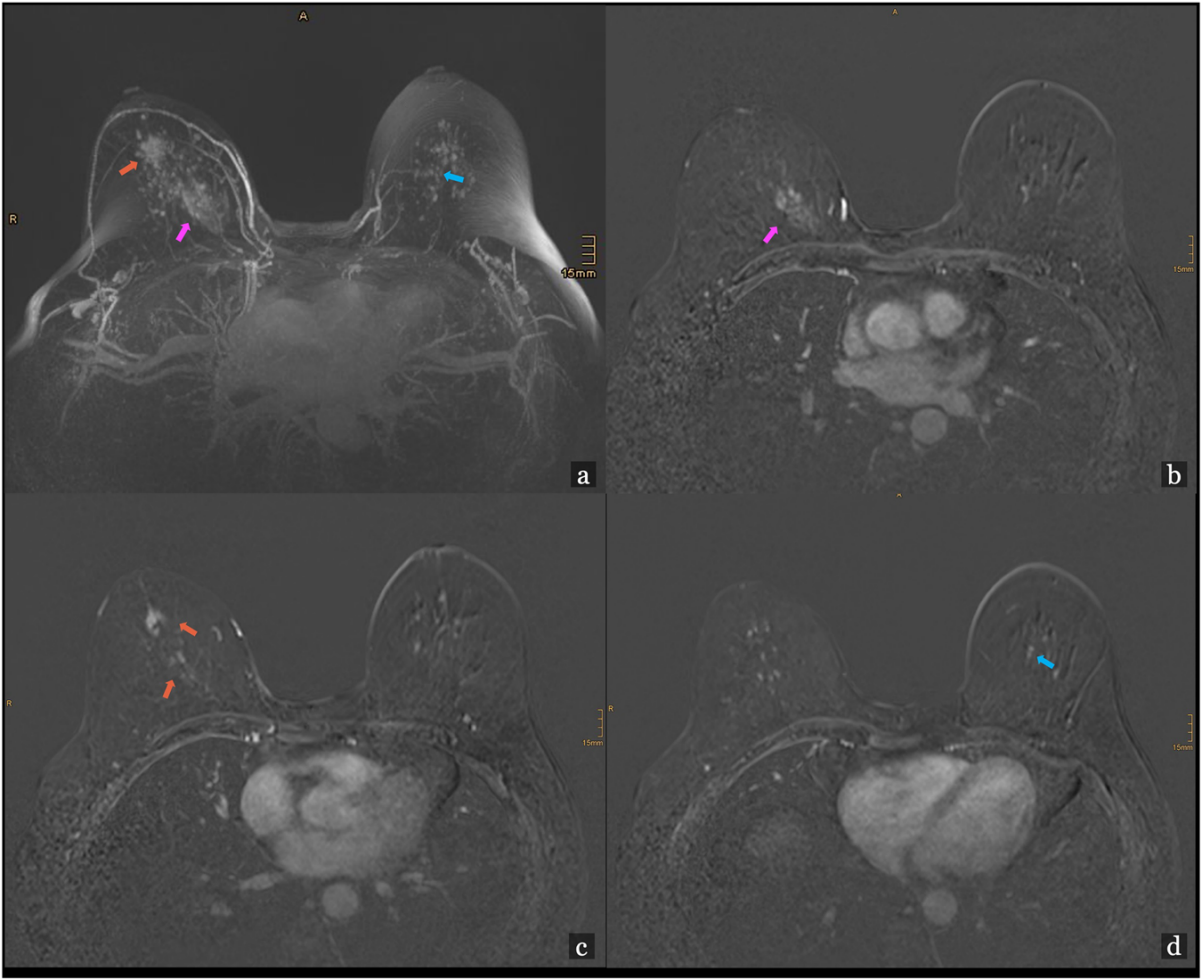
Woman, 65 years old, with ACR C and BPE moderate. **a** shows an MIP image with ROIs presenting significant findings. **b** In ROI 2 (pink arrow) depicts a 25 mm non-mass lesion assigned a BI-RADS 4 category (IDC with DCIS components); **c** ROI 5 (orange arrow) was assessed as a 45 mm BI-RADS 5 irregular mass (IDC); **d** ROI 9 (blue arrow) was assessed as a 7 mm BI-RADS 2 mass lesion (histology revealed tumor-free mammary parenchyma and microcalcification)

This may account for the observation that BPE did not hinder the assessment of the lesion by the readers [24]. The use of standardized tools, such as the Kaiser score, combined with a systematic assessment of the breast using an ROI-based approach, can improve diagnosis in breast lesions, even in cases of high BPE, thereby reducing the risk of both false-negative and false-positive results. In particular, adopting an ROI-based analytical approach enables precise detection and localization of multiple findings within the same patient [18, 25]. Because multiple ROIs were evaluated per case, this method can be considered more powerful than a solely patient-based assessment, which may overlook multifocal or multicentric malignancies as well as multiple false-positive enhancements (Fig. 6). ROI-level evaluation has been widely applied in imaging-based diagnostic studies research [26, 27] and provides a more stringent and granular measure of diagnostic performance by capturing false-positive and false-negative results that may coexist within a single patient.

A negative correlation between BPE levels and menopausal status [28] was found in this study. However, the effect of BPE on MRI performance was not influenced by hormonal status [29]. This may confer an advantage with respect to the logistical organization of MRI appointments, allowing for greater flexibility by omitting the regular timing currently observed in premenopausal women.

A recent study investigated the impact of BPE on cancer diagnosis, comparing DCE-MRI with contrast-enhanced mammography (CEM), and showed that there was no reduction in the diagnostic performance of MRI even with a moderate-marked BPE level, while there was a strong negative impact when CEM was used [30]. This phenomenon may be due to the two-dimensional nature of CEM, which makes it less useful than MRI for lesion detection in breasts with high BPE.

In the present study, the cancer prevalence was found to be similar in both BPE groups. This finding lends further support to the hypothesis that BPE levels should not be considered a measure of breast cancer risk, but rather as an expression of tissue activity [31].

In our study, the inter-reader agreement for the two BPE groups was moderate; this result is in line with the other prior studies [8, 32].

In our study, the readers were intentionally blinded to whether additional imaging modalities—such as mammography or ultrasound—had been performed as part of the clinical workflow, as well as to their respective findings. The potential diagnostic contribution offered by concomitant information from these techniques is well recognized and has been examined in previous investigations [33]. Nevertheless, in our cohort, the BPE level did not demonstrate any significant impact on the diagnostic performance metrics of breast MRI. Consequently, we did not incorporate or compare supplementary findings from mammography or ultrasound. For similar reasons, stratification according to potential confounders—such as age or menopausal status, FDG levels, underlying individual risk profile, or MRI field strength (1.5 T vs. 3 T)—was considered beyond the scope of the study. In particular, regarding the MRI field strength, prior prospective research [34] has shown comparable diagnostic accuracy across both field strengths, even though earlier studies [35] have reported that T1 relaxation times are longer at 3 T than at 1.5 T, meaning that the same dose of contrast agent could result in a more pronounced BPE on 3-T images than on 1.5-T images. This evidence indirectly supports our findings, indicating that such field strength–related variation does not meaningfully affect the ability of breast MRI to accurately detect malignant lesions, especially considering that both 1.5-T and 3-T systems are widely used in clinical practice.

This study has several limitations. It is a retrospective analysis involving a limited number of readers, which may restrict the generalizability of the findings. In our population, the subgroup with high BPE was relatively modest, as moderate and marked BPE were found in only 22% of patients. Potential preselection bias cannot be excluded. Besides, the level of BPE was only assessed by qualitative measurement. Nevertheless, there is a lack of evidence in the existing literature to support that there is a statistically significant difference between qualitative and quantitative data [32]. Moreover, the relevance of BPE as a marker of interpretation accuracy, cancer risk, and response to treatment remains unclear. Consequently, the value of automated assessment has yet to be proven. Another limitation is that the impact of these results on future diagnostic decisions is unclear since the short-term recall rates were not evaluated.

Finally, no systematic comparison was conducted to determine whether tools such as the Kaiser score would enhance diagnostic performance, either with or without the use of this approach.

## Conclusion

Our study demonstrates that BPE did not affect the diagnostic accuracy of DCE-MRI in detecting breast lesions. DCE-MRI can be used with confidence to diagnose breast cancer in different patient populations (e.g., with moderate and marked BPE) without a significant impact on diagnostic accuracy. The ROI-based approach may help both the detection and localization of multiple findings per patient.

## Supplementary information

ELECTRONIC SUPPLEMENTARY MATERIAL

## Abbreviations

AUC: Area under the curve
BPE: Background parenchymal enhancement
CEM: Contrast-enhanced mammography
DCE-MRI: Dynamic contrast-enhanced MRI
ROI: Region of interest
