# Supplementary material for: Impact of background parenchymal enhancement on the diagnosis of enhancing lesions in breast MRI: a systematic approach
Source: Eur Radiol. 2026 Apr 4;36(8):6669–78. doi: 10.1007/s00330-026-12474-y (PMC13342216; doi:10.1007/s00330-026-12474-y)
Supplement: Supplementary file 1 — ELECTRONIC SUPPLEMENTARY MATERIAL [file 330_2026_12474_MOESM1_ESM.pdf]

Impact of background parenchymal enhancement (BPE) on the diagnosis of enhancing lesions in breast MRI: a systematic approach

ELECTRONIC SUPPLEMENTARY MATERIAL

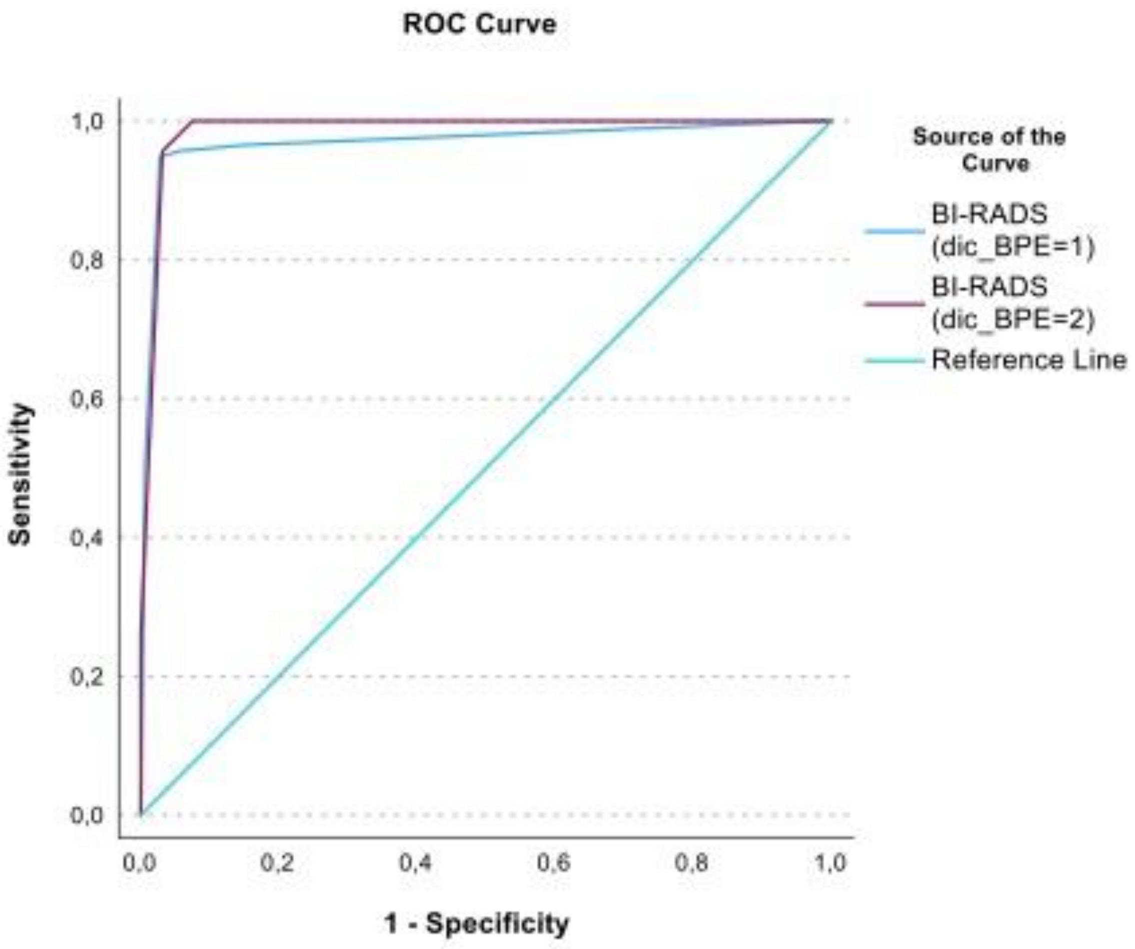

Supplement Figure 1. Performance of DCE-MRI in detecting breast lesions stratified per BPE groups.

Eur Radiol (2026) Santonocito A, Bechyna S, Clauser P, Helbich TH, Baltzer PAT.
